# 1457. Carbapenem-resistant *Acinetobacter baumannii* in an Intensive Care Unit during the COVID-19 pandemic: An Outbreak Investigation Utilizing Whole Genome Sequencing

**DOI:** 10.1093/ofid/ofad500.1294

**Published:** 2023-11-27

**Authors:** Samantha Williams, Rochelle T Thompson Kolawole, Bhagyashri Navalkele, Melinda Grubb, Sheila Fletcher, Daphne Ware, Ashley Robinson

**Affiliations:** University of Mississippi Medical Center, Lexington , South Carolina; University of Mississippi Medical Center, Lexington , South Carolina; University of Mississippi Medical Center, Lexington , South Carolina; The University of Mississippi Medical Center, Jackson, Mississippi; University of Mississippi Medical Center, Lexington , South Carolina; Mississippi State Department of Health, Jackson, Mississippi; University of Mississippi Medical Center, Lexington , South Carolina

## Abstract

**Background:**

Infections due to carbapenem-resistant *Acinetobacter baumannii* (CRAB) are associated with increased mortality, length of hospital stay, and cost. Due to the high patient volume during the Delta wave of the COVID-19 pandemic, one of the parking garages at our institution was converted into overflow ICU space that lacked institutional Infection Prevention (IP) oversight. Shortly afterwards, we experienced an outbreak of CRAB. Here we describe our outbreak investigation utilizing whole genome sequencing.

**Methods:**

After five cases of CRAB were noted in a ten-day period, enhanced IP measures were implemented, and an outbreak investigation was initiated (Figure 1). A hospital-onset (HO) CRAB case was defined as CRAB isolated from any specimen collected on or after hospital day three from August to September 2021 in patients without prior CRAB infection. A line list of HO CRAB cases was created. CRAB isolates underwent WGS and analysis (PA) (Figure 2). Location mapping was then performed on cases involved in the outbreak to assess potential transmission pathways.

**Figure:**
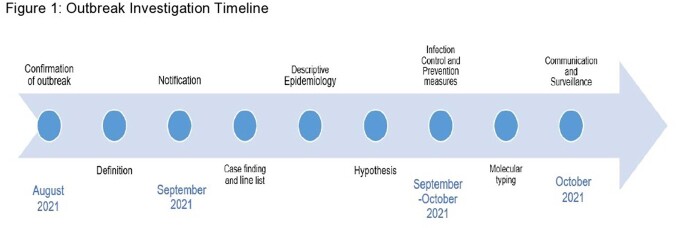

**Figure:**
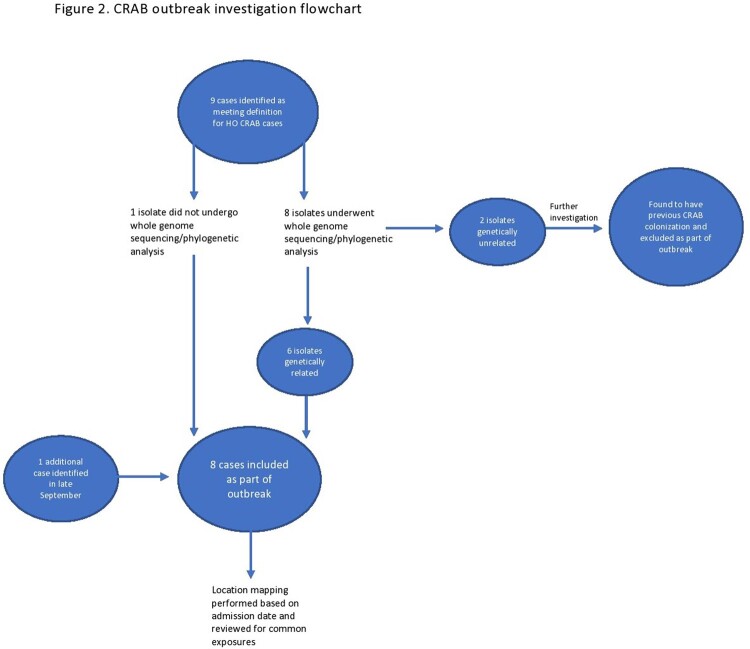

**Results:**

Nine cases of HO CRAB were initially identified. Eight of these underwent WGS and analysis (Figure 3). Based on WGS, six of the eight isolates were determined to be genetically related and were strains from global cluster 2 with intrinsic (OXA-51 family) and acquired (OXA-23) carbapenemases. Further investigation revealed the two unrelated isolates (C and H) had prior community-onset (CO) CRAB colonization, and they were excluded from the outbreak. One additional HO case was identified later during the outbreak and did not undergo WGS. A total of eight cases were labeled as HO CRAB. Location mapping of HO cases was performed. Multiple overlapping locations were noted among HO cases, with five of the eight cases notably having been located in the makeshift garage ICU during their stay (Figure 4). Patient demographics, clinical data, and outcomes are described in Table 1.

**Figure:**
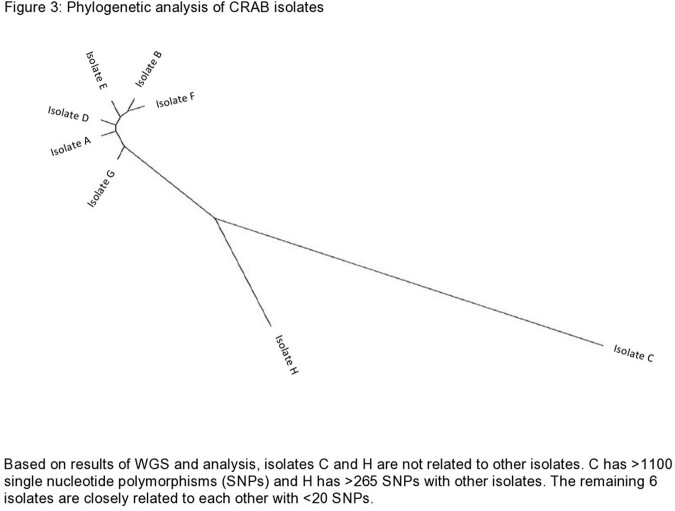

Based on results of WGS and analysis, isolates C and H are not related to other isolates. C has >1100 single nucleotide polymorphisms (SNPs) and H has >265 SNPs with other isolates. The remaining 6 isolates are closely related to each other with <20 SNPs.

**Figure:**
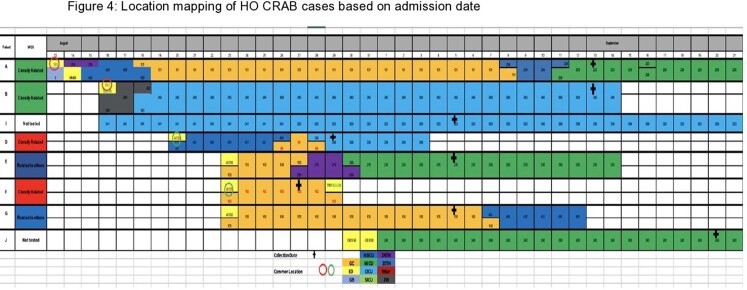

**Figure:**
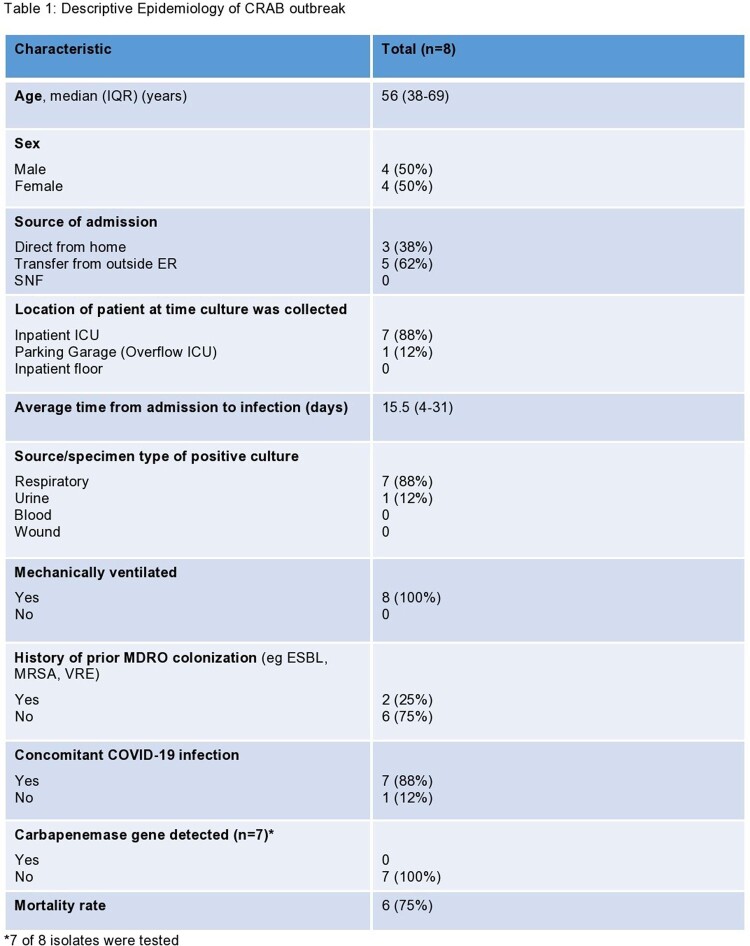

**Conclusion:**

Our study demonstrated the use of location mapping and utility of WGS to identify outlier cases and the strains involved in the CRAB outbreak. Our findings also highlight the need for IP involvement at the time of addition of new patient care areas to provide guidance on practices to help prevent outbreaks.

**Disclosures:**

**Ashley Robinson, PhD**, Pfizer: Grant/Research Support

